# Solid-state mechanochemical ω-functionalization of poly(ethylene glycol)

**DOI:** 10.3762/bjoc.13.191

**Published:** 2017-09-18

**Authors:** Michael Y Malca, Pierre-Olivier Ferko, Tomislav Friščić, Audrey Moores

**Affiliations:** 1 Department of Chemistry, McGill University, 801 Sherbrooke Street West, Montreal, QC, H3A 0B8, Canada

**Keywords:** amination, bromination, carboxylation, mechanochemistry, poly(ethylene glycol), solid state, thiolation, tosylation

## Abstract

Poly(ethylene glycol) (PEG) is a linear polymer with a wide range of applications in chemical manufacturing, drug development and nanotechnology. PEG derivatives are being increasingly used to covalently modify small molecule and peptide drugs, as well as bioactive nanomaterials in order to improve solubility in biological serum, reduce immunogenicity, and enhance pharmacokinetic profiles. Herein we present the development of mechanochemical procedures for PEG functionalization without the need for bulk solvents, offering a cleaner and more sustainable alternative to existing solution-based PEG procedures. The herein presented mechanochemical procedures enable rapid and solvent-free derivatization of PEG with tosyl, bromide, thiol, carboxylic acid or amine functionalities in good to quantitative yields and with no polymer chain oligomerization, proving the versatility of the method.

## Introduction

Poly(ethylene glycol) (PEG) is a linear polyether polymer with highly hydrophilic properties. Whereas PEG functionalization is restricted to its terminal functionalities, derivatization of these sites is essential for its use in pharmaceutical and material design. Specifically, modification of bioactive substrates with PEG is well established in drug development, and is also becoming important in the purification of proteins and nucleic acids [1]. Since the first demonstration of PEGylated proteins with altered immunogenicity [2–3], PEG has been heavily investigated for affording biologically active molecules with superior pharmacokinetic profiles and increased solubility in aqueous media [4–6]. A wide variety of modern PEGylated drugs take advantages of these properties: Mucagen (2004), Cimzia (2008) and Puricase (2010) are but a few examples [7]. On the other end, PEG is also being used to stabilize nanomaterials, allow their stable suspension in aqueous media, and interface them with biological systems [8–10]. Besides for its effects on solubility, PEG also creates a hydrodynamic barrier around the functionalized nanomaterial, allowing for reduced immunogenicity [11], leading to significant improvements in blood circulation half-lives, decrease in clearance rates, and prolonged pharmacological effects [12–14]. Derivatives of PEG are often used to perform conjugation reactions on small molecule drugs, proteins, or bioactive nanomaterials [15]. Other methods include chelation or ligand-exchange reactions at metal-based nanomaterials with ω-functionalized PEG polymers [16–18].

The two most common methods for accessing ω-functionalized PEG derivatives are solution-based through either ring-opening polymerization of ethylene oxide unites or modification of commercially available, parent hydroxy-terminated PEG [19]. The latter route is milder, more accessible and offers finer control over the polymer molecular weight. However, in both cases, the methods for PEG ω-functionalization raise concerns in terms of environmental impact, given that these reactions typically require dilute conditions under inert atmosphere, warranting large amounts of solvents and time [1,19–20]. High dilution during derivatization is a requirement of solvent-based syntheses to avoid unwanted chain lengthening caused by intermolecular reactions [21]. Having in mind the vocal demands of pharmaceutical industry for the development of cleaner, more efficient synthetic techniques [22], we now explore the possibility of accessing PEG derivatives in the solid-state. The use of mechanochemistry to achieve both supramolecular [23] and covalent [24] synthesis and modification of active pharmaceutical ingredients (APIs) is an emergent area that was recently reviewed [25]. In particular, solvent-free polymerization methods have been recently developed to access polyimines [26], polylactides [27], poly(phenylene vinylene) [28] and polyolefins [29]. There has been, however, limited effort towards the functionalization of premade polymers. Recently, Yan and co-workers used ball milling to deacetylate chitin to afford chitosan [30].

We now provide a proof-of-principle demonstration of mechanochemical ω-functionalization of α-protected methoxy-PEG (mPEG) with –COOH, –OTs, –NH_2_, –Br, and –SH functionalities, leading to rapid and cost-effective synthesis of these important derivatives in good to quantitative yields under aerobic conditions, using methoxypoly(ethylene glycol) of average molecular weights *M*_n_ = 750 Da and *M*_n_ = 2000 Da (mPEG_750_ and mPEG_2000_, respectively). We chose these derivatives because of their versatile applicability to covalent conjugation onto various substrates and metal-based nanomaterials.

## Results and Discussion

**Scheme 1:**
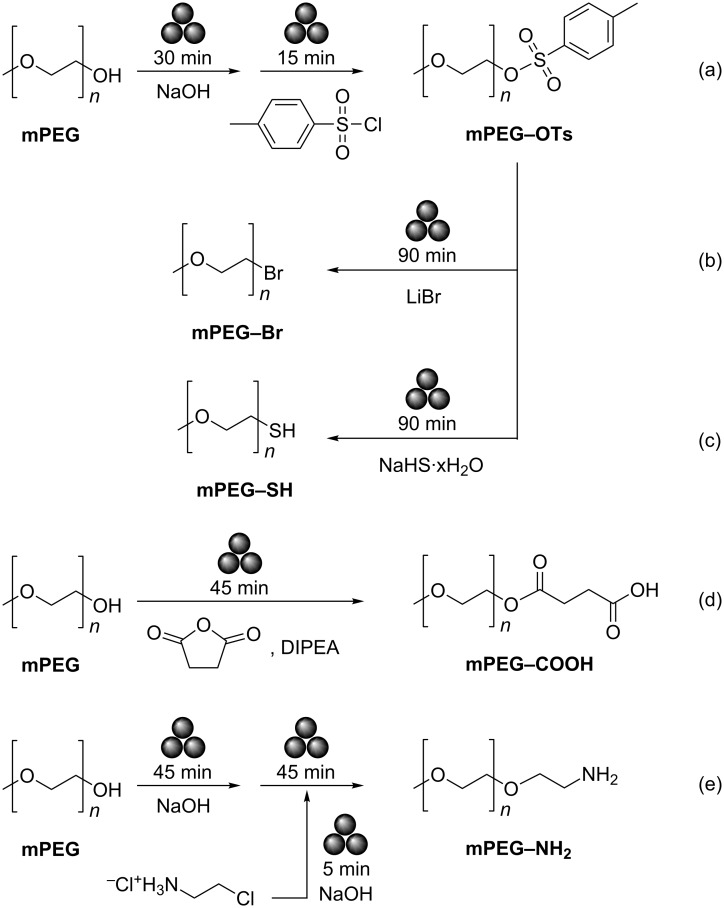
Developed syntheses for accessing by mechanochemistry: (a) mPEG–OTs, (b) mPEG–Br, (c) mPEG–SH, (d) mPEG–COOH, and (e) mPEG_x_–NH_2_. mPEG of *M*_n_ = 750 and 2000 Da were investigated as precursors. All milling reactions were performed at an operating frequency of 30 Hz.

For this study, we focused on the functionalization of mPEG, which allows the simple mono-functionalization of the polymer, for useful applications to drug development or nanomaterials (Scheme 1). To establish the generality of the method, we used mPEGs of two different, commercially available molecular weights, *M*_n_ = 750 and 2000 Da (mPEG_n_). In all the examples we explored in this study, reaction progress was determined by ^1^H NMR yields, where yields were determined by integration of peaks attributed to the methylene hydrogens geminal to the ω-functionality of mPEG, namely hydroxy, for the starting material, and the functionality introduced in the reaction explained below, for the products. *p*-Xylene was used as an internal standard for ^1^H NMR analysis, and the methoxy end of mPEG (singlet at 3.38 ppm) served to confirm conversions. Prudence was given to confirming interchain reactions did not occur by confirming mass balance in all cases.

**Figure 1:**
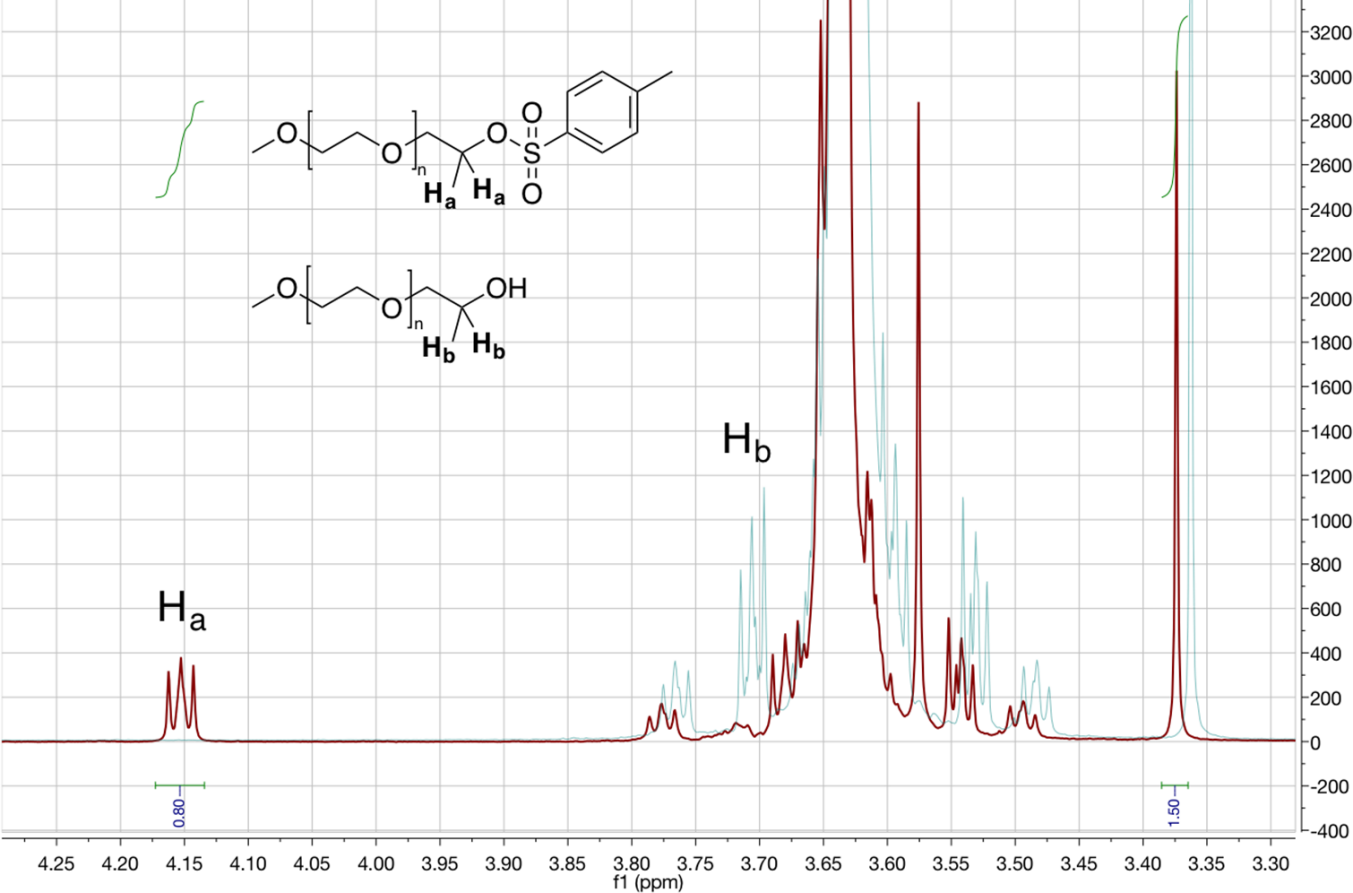
^1^H NMR of sample mPEG_2000_–OTs (Table 1, entry 5) in CDCl_3_ showing mPEG end group shift after tosylation.

We first explored the possibility of introducing a *p*-methylsulfonato (tosyl) moiety at the termination of mPEG by ball-milling. Namely, the tosyl moieties are known as excellent leaving groups, making tosylated mPEG (mPEG_x_–OTs) useful synthons for accessing further PEG derivatives. For this, we conducted a two-step one-pot reaction involving milling first the mPEG reactant with a base, followed by addition of *p*-toluenesulfonyl chloride (TsCl) and further milling (Scheme 1a, Table 1). mPEG_750_ was used to survey and optimize the tosylation reaction conditions. Milling of only mPEG with TsCl led to a poor conversion of 6% (Table 1, entry 1). However, addition of 1 equivalent of weak base, such as K_2_CO_3_ or *N*,*N*-diisopropylethylamine (DIPEA) led to ^1^H NMR yields of 21% and 17%, respectively (Table 1, entries 2 and 3). Switching to NaOH as the base led to a sharp increase of mPEG conversion to 81%. The highest conversions were obtained by using mPEG, NaOH and TsCl in respective stoichiometric ratios of 1:1.2:1.5 (Table 1, entry 4). These conditions functioned similarly with higher molecular weight mPEG_2000_ (Table 1, entry 5). In the ^1^H NMR spectra of these samples, the triplet of the terminal methylene moieties in the mPEG starting material at 3.72 ppm is replaced by one at 4.15 ppm, consistent with tosylation of the terminal group (Figure 1) [20]. The functionalization of mPEG was also corroborated by the observed shift in the ^1^H NMR signals of the tosylate group protons from 7.92 (2H) and 7.49 (2H) in TsCl to 7.79 and 7.34 ppm, in mPEG–OTs (Figure S1, Supporting Information File 1) [20].

**Table 1:** Surveyed reactions for mechanochemical derivatization of mPEG with tosylate functionality. TsCl = *p*-toluenesulfonyl chloride; CEA = chloroethylamine·HCl; *M*_w_ = molecular weight. All reactions were ball-milled at an operating frequency of 30 Hz.

Entry	mPEG *M*_w_	base (equiv)	TsCl (equiv)	Time (min)	^1^H NMR yield

**1**	750	–	1.2	45	6%
**2**	750	K_2_CO_3_ (1.0)	1.2	45	21%
**3**	750	DIPEA (1.0)	1.2	45	17%
**4**	750	NaOH (1.2)	1.5	15	81%
**5**	2000	NaOH (1.2)	1.5	15	80%

Employing NaOH as a base yielded the best results with both molecular weight (*M*_w_) mPEGs. NaOH is a strong base, thus favoring deprotonation of mPEG over weaker bases to facilitate subsequent tosylation. The deprotonation step (Scheme 1a) also generates water locally, which may have led to liquid-assisted grinding (LAG) conditions and facilitated the interaction and mobility of substrates [31–33], and allowed the substrates to better interact in situ. Given that DIPEA did not afford high yields albeit being liquid and having a p*K*_a_ of 3.02, smaller than the one of mPEG (p*K*_a_ = 4.5–4.8), it suggests that solvation may play a role in promoting the reaction. Interestingly TsCl is prone to hydrolysis in the presence of water, yet it did not seem to affect the high reactivity observed with NaOH, likely because the generated, strongly nucleophilic, alkoxide would react even faster.

Progress of reactions in entries 4 and 5 in Table 1 was probed every 15 minutes at the second step (Scheme 1a). After 15 minutes milling, the reaction was complete, as almost identical ^1^H NMR yields were obtained for up to 75 min milling for both mPEG_750_ and mPEG_2000_.

The mechanochemically prepared tosylated polymers provided an entry into the synthesis of other mPEG derivatives by mechanochemistry, through ball-milling reaction with additional nucleophiles. The synthesis of terminally brominated mPEG (mPEG–Br) derivatives was achieved by milling of mPEG–OTs with LiBr (Scheme 1b). Analysis of the milled reaction mixture by ^1^H NMR revealed the appearance of a new triplet resonance centered at about 3.45 ppm in CDCl_3_, consistent with the methylene germinal to Br in mPEG–C*H*_2_-Br (Figure S2, Supporting Information File 1) [34–35]. ^1^H NMR yields of 58% and 65% were obtained for reactants mPEG_750_ and mPEG_2000_, respectively (Table 2, entries 1 and 2). 2D-HSQC was performed to validate terminal bromo functionality showing a cross-peak at ^1^H, ^13^C = 3.45 ppm, 30.10 ppm (Figure S3, Supporting Information File 1). These results are exciting given that PEG bromination is often performed under harsh conditions either via radical intermediates or using bromoacyl halides, which introduces unnecessary ester groups instead of direct bromine substitution onto the polymer chain [34,36–37].

**Table 2:** Surveyed reactions of mechanochemical derivatization to afford mPEG–Br, –SH, –COOH and –NH_2_ derivatives.

Entry	Product	Time of milling (min)	^1^H NMR yield

**1**	mPEG_750_-Br	90	58%
**2**	mPEG_2000_-Br	90	65%
**3**	mPEG_750_-SH	90	48%^a^
**4**	mPEG_2000_-SH	90	69%^a^
**5**	mPEG_750_-COOH	45	99%
**6**	mPEG_2000_-COOH	45	90%
**7**	mPEG_750_-NH_2_	45	42%
**8**	mPEG_2000_-NH_2_	45	63%

Reaction conditions for entries 6 and 7: mPEG–OTs, LiBr (3 equiv); for entries 8 and 9: mPEG–OTs, NaHS·xH_2_O (2 equiv assuming 3 H_2_O); for entries 10 and 11: mPEG, DIPEA (0.2 equiv), succinic anhydride (1.2 equiv); for entries 12 and 13: mPEG, NaOH (1.2 equiv), CEA·HCl/NaOH (1.2 equiv). All reactions were ball-milled at an operating frequency of 30 Hz. ^a^Corresponding disulfides were also observed as minor side product.

Next, we explored the thiolation by milling the mPEG–OTs with NaHS·xH_2_O for 90 min (Table 2, entries 3 and 4) as reagent, which afforded ^1^H NMR conversions of 55% and 78% for *M*_n_ = 750 and 2000 Da, respectively. In this reaction, thiol was obtained as major product, with a small portion of disulfide as byproduct. Yield of 48% −SH + 7% –S–S– and 69% –SH + 9% –S–S– were measured for *M*_n_ = 750 and 2000 Da, respectively. In the ^1^H NMR spectra, the mPEG–SH was clearly identified by a triplet at 2.86 ppm, characteristic of methylene hydrogens germinal to thiol, while the corresponding peak of mPEG–S–S–mPEG appeared at 2.72 ppm (Figure S4, Supporting Information File 1) [20]. The formation of the disulfide derivatives is explained by the reaction being performed under aerobic conditions [20].

To access mPEG–carboxylate (mPEG–COOH) under milling conditions, native mPEG was reacted directly with succinic anhydride in the presence of catalytic amounts of DIPEA (Scheme 1d; Table 2, entries 5 and 6). Quantitative yields (>99%) of the mPEG_750_–COOH were obtained after only 45 min of milling (Figure S5, Supporting Information File 1) [38]. The end hydroxy group of mPEG at 3.72 disappeared and was replaced by a peak at 4.23 ppm after carboxy functionalization, further proving that the reaction was successful. The starting material succinic anhydride featured a singlet at 3.01 ppm, while the open structure resulting from the reaction with mPEG is characterized by two triplets centered at 2.54 and 2.62 ppm (Figure S6, Supporting Information File 1) [38]. The reaction was readily adaptable to the mPEG_2000_ reactant, in 90% yield according to ^1^H NMR spectroscopy.

Finally, we explored the possibility of accessing mPEG–NH_2_ polymers by using chloroethylamine hydrochloride (CEA·HCl) as an aminating agent (Scheme 1e). For this purpose, both mPEG and CEA·HCl were reacted separately mechanochemically with NaOH to afford the deprotonated mPEG and CEA free base, respectively. CEA·HCl was milled with NaOH briefly for only 5 min to avoid polymerization of the free base before reaction with mPEG. The milled products were then mixed and milled for 45 minutes, leading to a yield of 42% and 63% (for *M*_n_ = 750 and 2000 Da, respectively), according to ^1^H NMR spectroscopy (Table 2, entries 7 and 8). Analysis by ^1^H NMR revealed a new triplet at 2.98 ppm, characteristic of the methylene hydrogens germinal to NH_2_ (Figure S7, Supporting Information File 1) [20,39]. A 2D-HSQC measurement was performed to validate the addition of this functionality at the terminus of mPEG, showing a cross-peak at (^1^H, ^13^C) = (3.98 ppm, 43.63 ppm) (Figure S8, Supporting Information File 1) [20,39].

Importantly, in all the samples studied for this reaction, complete mass balance was obtained, using an external standard and the ^1^H NMR signal of the terminal methoxy group of mPEG. This allowed to establish that unfunctionalized polymers were all recovered after reaction as unreacted mPEG and not as mPEG dimers resulting from the intermolecular coupling of two chains. Interestingly, in solvent-based synthesis, dilute conditions are typically required to avoid intermolecular reactions between chains leading to unwanted chain lengthening during the derivatization process. Under mechanochemical conditions, diffusion limitation may favor the reactivity of small molecule reagents over the intermolecular reaction between two polymers to afford the kinetically-favorable end-products, in contrast to solvent-based conditions [21].

## Conclusion

We have demonstrated the rapid, efficient and selective synthesis of various PEG derivatives under mechanochemical conditions, without using any bulk solvent. The short times required to achieve reaction completion (45–90 minutes) contrast with the often several hour-long solvent-based reaction conditions [19,40]. Our results also show that solvent-free conditions for the post-functionalization of native PEG is a good avenue to prevent chain lengthening, a known limitation of solvent-based techniques. Finally, our method is advantageous over solvent-based ones, as it eliminates the need for inert atmosphere. Overall, the excellent reactivity and selectivity in the absence of bulk solvent is, to the best of our knowledge, unprecedented.

## Supporting Information

File 1 Experimental part and NMR spectra.
