# Supplementary material for: Solid-state mechanochemical ω-functionalization of poly(ethylene glycol)
Source: Beilstein J Org Chem. 2017 Sep 18;13:1963–8. doi: 10.3762/bjoc.13.191 (PMC5629410; doi:10.3762/bjoc.13.191)
Supplement: File 1 — Experimental part and NMR spectra. [file Beilstein_J_Org_Chem-13-1963-s001.pdf]

Supporting Information

for

Solid-state mechanochemical ω-functionalization of poly(ethylene glycol)

Michael Y. Malca, Pierre-Olivier Ferko, Tomislav Friščić and Audrey Moores*

Address: Department of Chemistry, McGill University, 801 Sherbrooke Street West, Montreal, QC, H3A 0B8, Canada

Email: Audrey Moores - audrey.moores@mcgill.ca

*Corresponding author

Experimental part and NMR spectra

Materials and instrumentation

Poly(ethylene glycol) methyl ether (M_n = 750 and 2000 Da) ($mPEG_x$), *p*-toluenesulfonyl chloride (TsCl, ≥98%), diisopropylamine (DIPEA, ≥99%), succinic anhydride (97%), sodium hydrosulfide hydrate ($NaHS{\cdot}xH_2O$), lithium bromide (LiBr, ≥99%), chloroethylamine hydrochloride (CEA, 99%) and *p*-xylene (anhydrous, ≥99%) were purchased from Sigma-Aldrich. Sodium hydroxide pellets (NaOH, 97%) were purchased from ACS reagents. Potassium carbonate (K_2CO_3 anhydrous, ≥99%) was purchased from EMD Millipore. 10 mL Teflon jars and 10 mm Zr balls were obtained from FORM-TECH Scientific. The milling reactions were performed using a Retsch Mixer Mill 400 at an operating frequency of 30 Hz. ^{1}H NMR and 2D-HSQC spectra were recorded using a Bruker AV500 operating at 500 MHz.

Methods

Synthesis of $mPEG_{750}$-OTs: 150.0 mg mPEG (M_n - 750 g/mol) and 9.6 mg NaOH (1.2 equiv) were loaded in a 10 mL Teflon jar with one 10 mm Zr ball and milled for 30 min at 30 Hz with a drop of *p*-xylene as an internal standard. Alternatively, 27.64 mg K_2CO_3 (1.0 equiv) was added as the base. 57.20 mg TsCl (1.5 equiv) were then added to the reaction vessel and milled for 15–45 min at 30 Hz. The crude product was then dissolved in $CDCl_3$ and filtered through a Celite/cotton plug.

^{1}H NMR: δ 7.33 (d, J = 8.1 Hz); 4.14 (t, J = 5.1 Hz)

Synthesis of $mPEG_{2000}$-OTs: 150.0 mg mPEG (M_n = 2000 g/mol) and 3–4.5 mg NaOH (1.0–1.5 equiv) were loaded in a 10 mL Teflon jar with one 10 mm Zr ball and milled for 30 min at 30 Hz with a drop of *p*-xylene as an internal standard. 21.45 mg TsCl (1.5 equiv) were then added to the reaction vessel and milled for 15–45 min at 30 Hz. The crude product was then dissolved in $CDCl_3$ and filtered through a Celite/cotton plug. ^{1}H NMR: δ 7.34 (d, J = 8.1 Hz); 4.15 (t, J = 4.8 Hz)

Synthesis of $mPEG_{750}$-Br: 150.0 mg PEG_{955}-OTs and 40.92 mg LiBr (3 equiv) were loaded in a 10 mL Teflon jar with one 10 mm Zr ball and milled for 90 min at 30 Hz with a drop of *p*-xylene as an internal standard. The crude product was then dissolved in $CDCl_3$ and filtered through a Celite/cotton plug. ^{1}H NMR: δ 3.37 (s); 3.47 (t, J = 6.3 Hz); 3.50 (t, J = 5.8 Hz); 3.69 (t, J = 5.8 Hz); 3.80 (t, J = 6.3 Hz).

Synthesis of $mPEG_{2000}$-Br: 150.0 mg PEG_{2155}-OTs and 18.13 mg LiBr (3 equiv) were loaded in a 10 mL Teflon jar with one 10 mm Zr ball and milled for 90 min at 30 Hz with a drop of *p*-xylene as an internal standard. The crude product was then dissolved in $CDCl_3$ and filtered through a Celite/cotton plug. ^{1}H NMR: δ 3.36 (s); 3.45 (t, J = 6.3 Hz); 3.79 (t, J = 6.4 Hz). 2D-HSQC: δ (3.47, 30.10) corresponding to -O-$CH_2\underline{CH_2}$-Br

Synthesis of $mPEG_{750}$-SH: 150.0 mg PEG_{955}-OTs and 44 mg NaHS·xH_2O (2 equiv assuming 3 H_2O) were loaded in a 10 mL Teflon jar with one 10 mm Zr ball and milled for 90 min at 30 Hz with a drop of *p*-xylene as an internal standard. The crude product was then dissolved in $CDCl_3$ and filtered through a Celite/cotton plug. ^{1}H NMR: δ 2.72 (t, *J* = 6.9 Hz); 2.86 (t, *J* = 6.8 Hz); 3.35 (s); 3.48 (t, *J* = 5.8 Hz); 3.76 (t, *J* = 5.9 Hz).

Synthesis of $mPEG_{2000}$-SH: 150.0 mg PEG_{2155}-OTs and 16.5 mg NaHS·xH_2O (2 equiv assuming 3 H_2O) were loaded in a 10 mL Teflon jar with one 10 mm Zr ball and milled for 90 min at 30 Hz with a drop of *p*-xylene as an internal standard. The crude product was then dissolved in $CDCl_3$ and filtered through a Celite/cotton plug. ^{1}H NMR: δ 2.72 (t, *J* = 6.9 Hz); 2.86 (t, *J* = 6.7 Hz); 3.36 (s); 3.48 (t, *J* = 5.8 Hz); 3.76 (t, *J* = 5.8 Hz).

Synthesis of $mPEG_{750}$-COOH: 150.0 mg of mPEG (M_n = 750 g/mol), 5.2 mg DIPEA (0.2 equiv) and 24.0 mg succinic anhydride (1.2 equiv) were loaded in a 10 mL Teflon jar with one 10 mm Zr ball and milled using a Retsch Mixer Mill 400 in air for 45 min at 30 Hz. A drop of *p*-xylene was added to the reaction vessel as an internal standard. The crude product was then dissolved in $CDCl_3$ and filtered through a Celite/cotton plug. ^{1}H NMR: δ 4.21 (t, *J* = 4.9 Hz); 2.62 (t, *J* = 7.0 Hz); 2.54 (t, *J* = 7.0 Hz).

Synthesis of $mPEG_{2000}$-COOH: 150.0 mg of mPEG (M_n = 750 g/mol), 5.2 mg DIPEA (0.2 equiv) and 24.0 mg succinic anhydride (1.2 equiv) were loaded in a 10 mL Teflon jar with one 10 mm Zr ball and milled using a Retsch Mixer Mill 400 in air for 45 min at 30 Hz. A drop of *p*-xylene was added to the reaction vessel as an internal standard. The crude product was then dissolved in $CDCl_3$ and filtered through a Celite/cotton plug. ^{1}H NMR: δ 4.21 (t, *J* = 4.9 Hz); 2.62 (t, *J* = 7.0 Hz); 2.54 (t, *J* = 7.0 Hz).

Synthesis of $mPEG_{750}$-NH_2: 150.0 mg of mPEG (M_n = 750 g/mol) and 9.6 mg NaOH (1.2 equiv) were loaded in a 10 mL Teflon jar with one 10 mm Zr ball and milled for 45 min at 30 Hz. In a separate jar, 27.84 mg CEA-HCl (1.2 equiv) and 9.6 mg NaOH were loaded in a 10 mL Teflon jar

with one 10 mm Zr ball and milled for 5 min at 30 Hz. The contents of the first step were added to the second jar with a drop of *p*-xylene as an internal standard, and the reaction was milled for 45 min at 30 Hz. The crude product was then dissolved in $CDCl_3$ and filtered through a Celite/cotton plug. ^1H NMR: δ 2.97 (t, J = 5.6 Hz); 3.35 (s); 3.47 (t, J = 5.9 Hz); 3.75 (t, J = 5.9 Hz).

Synthesis of mPEG$_{2000}$-NH$_2$: 150.0 mg of mPEG (M_n = 750 g/mol) and 3.6 mg NaOH (1.2 equiv) were loaded in a 10 mL Teflon jar with one 10 mm Zr ball and milled for 45 min at 30 Hz. In a separate jar, 10.44 mg CEA-HCl (1.2 equiv) and 3.6 mg NaOH were loaded in a 10 mL Teflon jar with one 10 mm Zr ball and milled for 5 min at 30 Hz. The contents of the first step were added to the second jar with a drop of *p*-xylene as an internal standard, and the reaction was milled for 45 min at 30 Hz. The crude product was then dissolved in $CDCl_3$ and filtered through a Celite/cotton plug. ^1H NMR: δ 2.98 (t, J = 5.6 Hz); 3.35 (s); 3.47 (t, J = 5.9 Hz); 3.76 (t, J = 5.9 Hz). 2D-HSQC: δ (2.98, 46.63) corresponding to -O-CH$_2$$\underline{CH_2}$-NH$_2$.

NMR characterization:

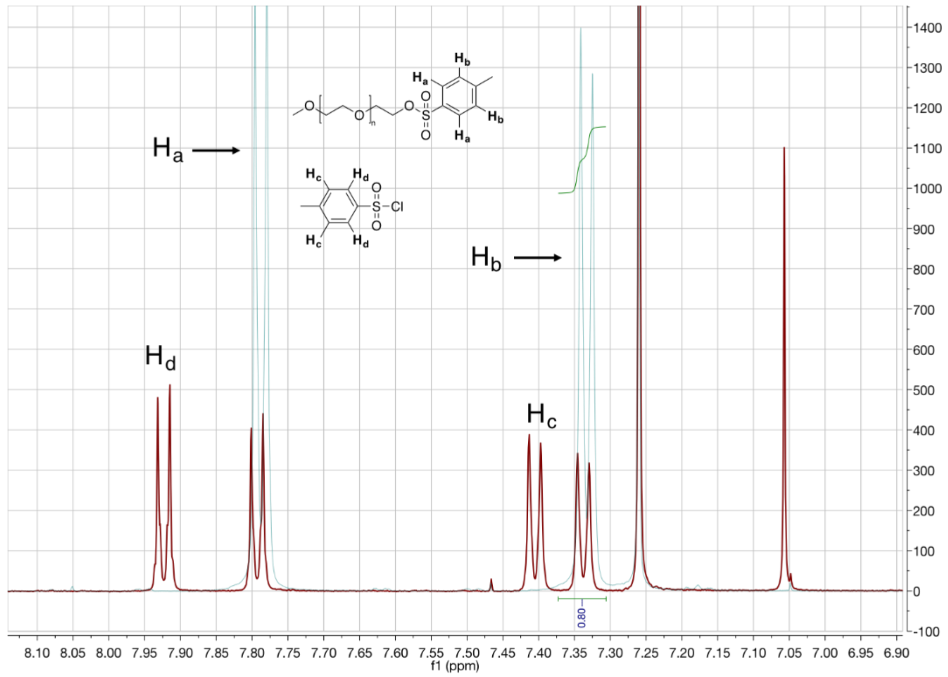

Figure S1: Comparison of tosylated PEG (red) and TsCl starting material (blue). Some TsCl starting material can be seen in the product spectrum.

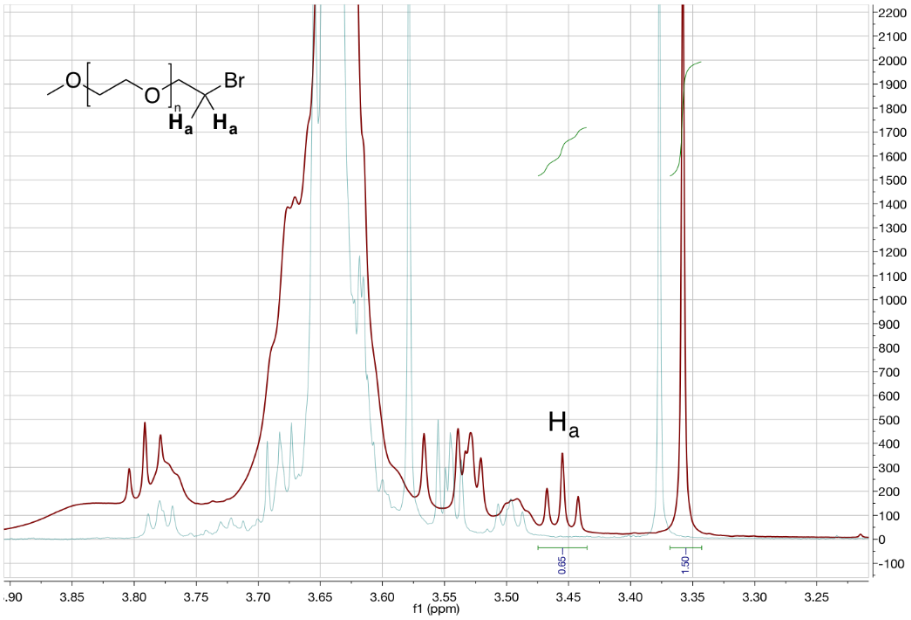

Figure S2: ^{1}H NMR of synthesized mPEG$_{2000}$-Br showing the appearance of the triplet centered at 3.45 ppm corresponding to $\text{-O-CH}_2\text{C}\underline{\text{H}_2}\text{-Br}$.

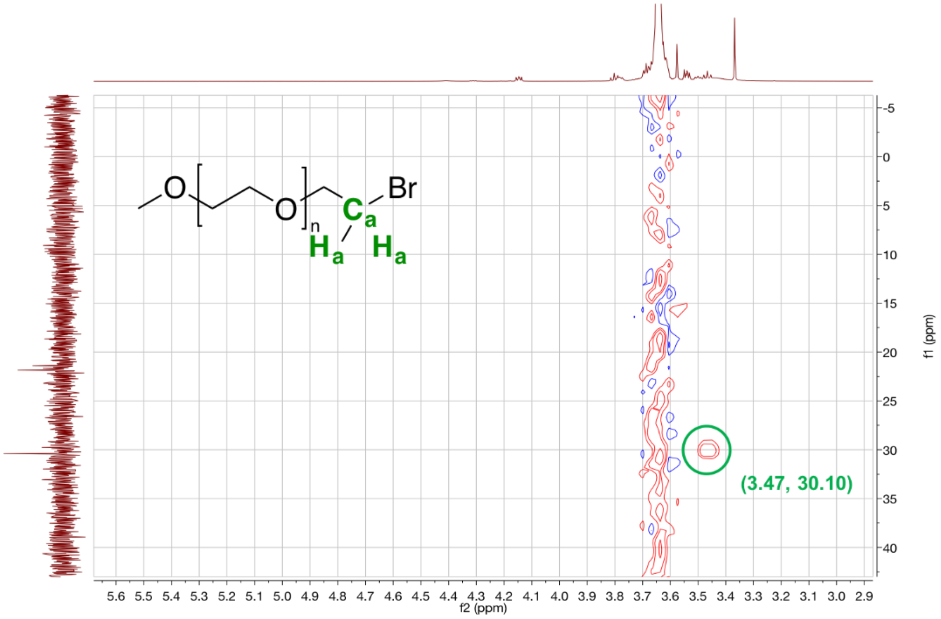

Figure S3: 2D-HSQC of synthesized mPEG$_{2000}$-Br showing the appearance of the correlated peak at (x,y) = (3.47, 30.10) corresponding to $\text{-O-CH}_2\underline{\text{CH}_2}\text{-Br}$.

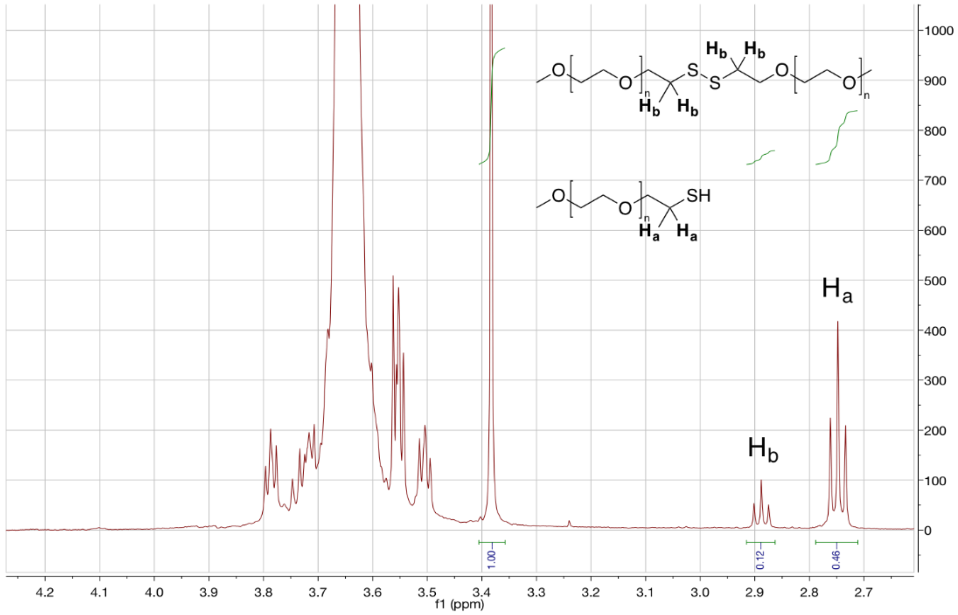

Figure S4: ^{1}H NMR of synthesized $mPEG_{2000}$-SH showing the appearance of the two triplets centered at 2.72 and 2.86 ppm corresponding to $-O-CH_2C\underline{H_2}-SH$ and $-O-CH_2C\underline{H_2}-S-S-C\underline{H_2}CH_2-O-$, respectively.

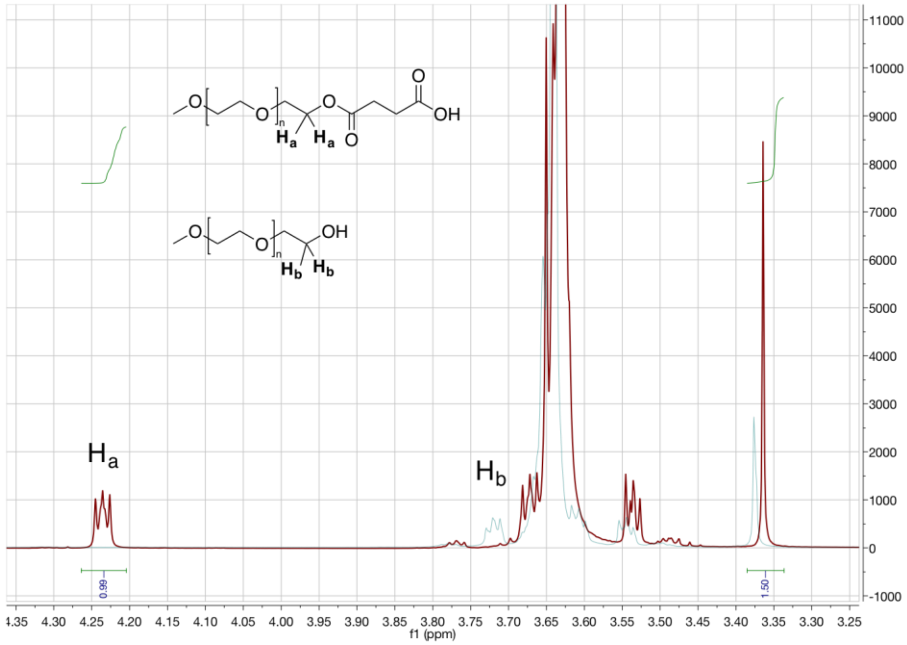

Figure S5: ^{1}H NMR of $mPEG_{750}$-COOH in $CDCl_3$ showing the mPEG end group shift from 3.72 to 4.23 ppm.

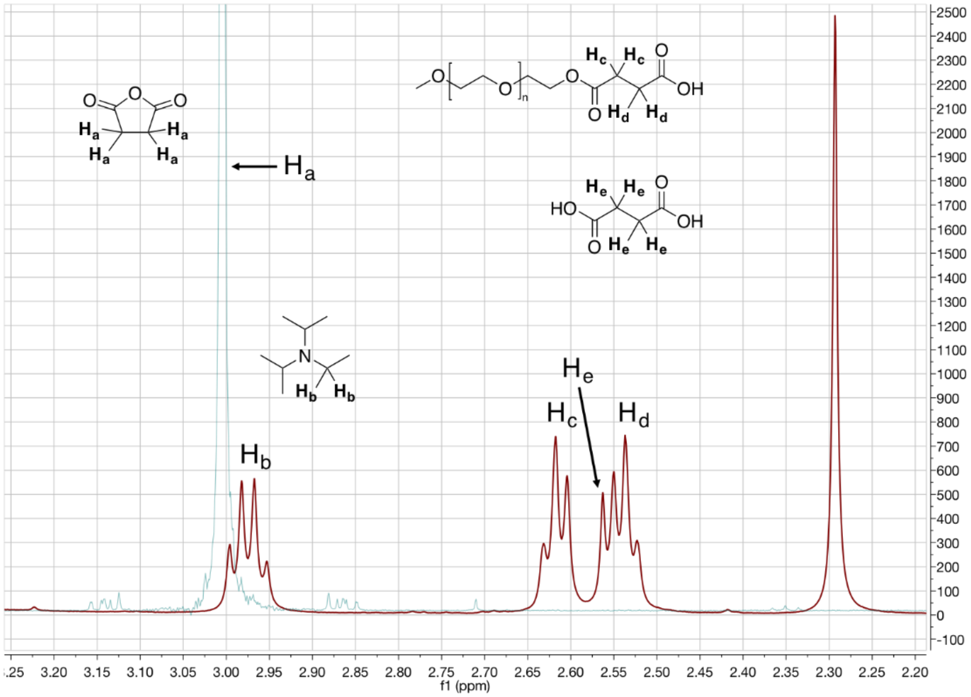

Figure S6: ^{1}H NMR showing appearance of the conjugated PEG-COOH (red) and succinic anhydride starting material (blue). Free succinate and DIPEA can be seen in the spectrum.

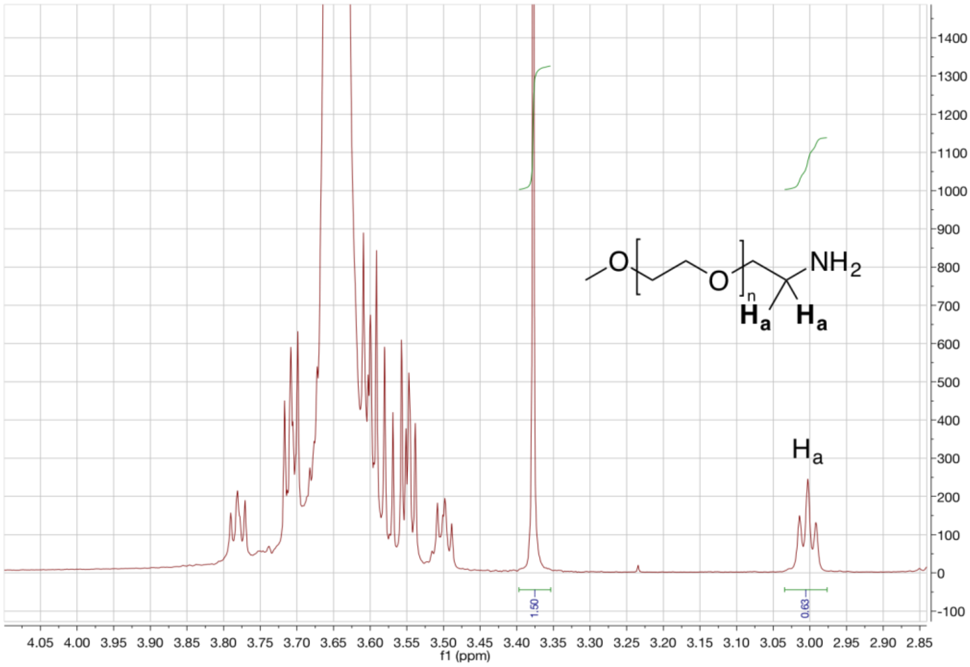

Figure S7: ^{1}H NMR of synthesized $mPEG_{2000}$-NH_2 showing the appearance of a triplet centered at 2.98 ppm corresponding to $-O-CH_2C\underline{H_2}-NH_2$.

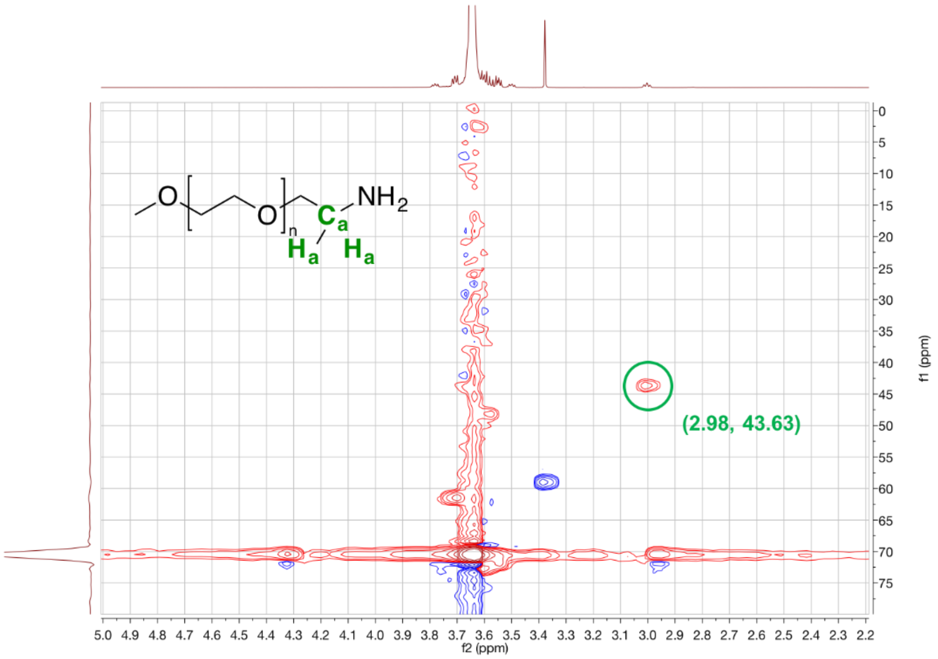

Figure S8: 2D-HSQC of synthesized $mPEG_{2000}$-Br showing the appearance of the correlated peak at (x,y) = (2.98, 43.63) (red) corresponding to -O-$CH_2\underline{CH_2}$-NH_2.
